# Anti-Metastatic Effects of Antrodan with and without Cisplatin on Lewis Lung Carcinomas in a Mouse Xenograft Model

**DOI:** 10.3390/ijms19061565

**Published:** 2018-05-24

**Authors:** Pei-Chun Chen, Chin-Chu Chen, Yaw-Bee Ker, Chi-Huang Chang, Charng-Cherng Chyau, Miao-Lin Hu

**Affiliations:** 1 Department of Food Science and Biotechnology, National Chung Hsing University, 250 Kuo Kuang Road, Taichung 402, Taiwan; baopeipei@gmail.com (P.-C.C.); mlhuhu@nchu.edu.tw (M.-L.H.); 2 Grape King Biotechnology Center, 60, Sec 3, Longgang Rd., Chung-Li City, Taoyuan County 320, Taiwan; gkbioeng@grapeking.com.tw; 3 Department of Food Science and Technology, Hungkuang University, No. 1018, Sec. 6, Taiwan Boulevard, Shalu District, Taichung City 43302, Taiwan; ybker@sunrise.hk.edu.tw; 4 Research Institute of Biotechnology, Hungkuang University, No. 1018, Sec. 6, Taiwan Boulevard, Shalu District, Taichung City 43302, Taiwan; ok1456@sunrise.hk.edu.tw

**Keywords:** *Antrodia cinnamomea*, antrodan, Lewis lung carcinoma, metastasis, MMPs, STAT3, MAPK, ERK, JNK, p38, immunity

## Abstract

Antrodan, a unique protein-bound polysaccharide derived from the fungal mycelia of *Antrodia cinnamomea*, has been reported to exhibit antitumor and anti-metastatic effects on Lewis lung carcinoma (LLC) cells through direct action and immunomodulation in vitro. In this study, we investigated the combined treatment of antrodan with an anti-cancer drug—cisplatin—and its underlying molecular mechanisms of action in a mouse xenograft tumor model. C57BL/6 mice were implanted (s.c.) with LLCs for nine days, before administration with only antrodan (20 mg/kg and 40 mg/kg; p.o.) daily, only cisplatin (1 mg/kg; i.p.) twice per week, or a combination of both for an additional 28 days. As expected, antrodan on its own significantly inhibited metastasis of lung and liver tissues, while treatment with cisplatin only merely inhibited metastasis of the liver. Antrodan exhibited efficient adjuvant therapy in combination with cisplatin, by inhibiting the activities of the plasma urokinase plasminogen activator (uPA) and the liver matrix metalloproteinase 9 (MMP-9), as well as by inhibiting the phosphorylation of p38 and extracellular signal-regulated kinase 2 (ERK2) in lung and liver tissues. In addition, antrodan effectively ameliorated cisplatin-induced kidney dysfunction when treated combinatorially, as evidenced by a decrease in cisplatin-induced blood urea nitrogen (BUN) levels in plasma and in the level of p38 phosphorylation in the kidney. Mechanistically, the actions of antrodan on its own involved (i) reducing the activities of uPA and MMP-2 and -9 in plasma; (ii) reducing protein expression of MMP-2/9, and the phosphorylation of signal transducer and activator of transcription 3 (STAT3) and mitogen-activated protein kinases (MAPKs), including extracellular signal-regulated kinases (ERKs), c-Jun N-terminal kinases (JNKs), and p38 in lung and liver tissues; and (iii) enhancing immune system functions resulting in the promotion of an anti-metastatic response through immunomodulation, by increasing interferon-γ (IFN-γ) levels and decreasing interleukin-6 (IL-6) levels in plasma. These results demonstrated that antrodan provides a novel, complementary therapeutic strategy against cancer metastasis, by attenuating the activities of MMP-2 and -9 through the modulation of STAT3/MAPK/ERK/JNK signaling pathways, and of the host’s immune system.

## 1. Introduction

Metastatic cancers are one of the leading causes of death. Statistical data indicate that tumor metastasis is responsible for approximately 90% of all cancer-related deaths [1]. The metastasis process consists of a complex cascade of events, including angiogenesis, migration, and invasion. In the tumor microenvironment, matrix metalloproteinases (MMPs) degrade the extracellular matrix, and secrete the vascular endothelial growth factor (VEGF) responsible for providing nutrients to cancer cells, and for inducing vascular proliferation. These tumor cells may undergo migration and invasion, traveling through the blood or lymphatic vessels, eventually leaving the blood or lymphatic vessels, before adhering to and growing at a distal site [2,3].

Among the biomarkers of cancer metastasis, MMPs, a family of zinc-dependent enzymes, play an important role in many physiological functions, such as embryonic development, wound healing, angiogenesis, tissue remodeling, and the regulation of inflammatory responses [4,5]. MMP-2 and MMP-9 are highly expressed in malignant tumors with a high metastatic capacity. These proteinases are considered the key factors contributing to cancer cell invasion and cancer metastasis [6]. The formation of active MMPs may be linked to effects caused by the urokinase plasminogen activator (uPA). uPA, a serine protease, is often overexpressed in non-small-cell lung cancers, liver cancers, colorectal cancers, and breast cancers, and is recognized as being responsible for poor prognoses and cancer metastasis [7,8,9]. In the tumor microenvironment, signal transducer and activator of transcription (STAT) proteins also play a vital role in promoting or inhibiting tumor metastasis. STAT3 proteins have been reported to downregulate expression of the vascular endothelial growth factor (VEGF), thereby reducing angiogenesis and delaying progress of the tumor [10]. As the STAT3 pathway is mainly responsible for the regulation of cell proliferation, invasion, and angiogenesis, many reports have shown that its sustained activation promotes the metastasis of many cancers, including thymic tumors, colorectal cancers, and squamous cell carcinomas of the skin. Thus, the STAT3 pathway is thought to be associated with cancer metastasis, and the suppression of this pathway can be used as one of the strategies in cancer therapy [11,12]. In addition to the STAT3 pathway, related transcription factors named mitogen-activated protein kinases (MAPKs) are involved in cell proliferation, apoptosis, angiogenesis, migration, and metastasis. The MAPK pathway can be divided into three subclasses: extracellular signal-regulated kinases 1 and 2 (ERK1/2), c-Jun N-terminal kinases (JNKs), and p38 pathways. The ERK1/2-MAPK pathway can be activated in a number of ways, including through growth factors, cytokines, viral infections, G-coupled protein receptors, and carcinogenic factors [13]. JNK proteins regulate three genes—MAPK8, MAPK9, and MAPK10—which have been found to produce JNK1, JNK2, and JNK3 proteins, respectively. JNK1 and JNK2 are expressed in most cells, while JNK3 is mainly present in the brain. JNKs primarily affect the expression of the AP1 transcription factor. The oncogenic functions of JNKs are mostly based on their ability to phosphorylate Jun, and to activate AP1, thus promoting cancer cell growth [14].

Cisplatin, one of many well-known anti-cancer drugs, was approved for cancer treatment by the Food and Drug Administration (FDA) in 1979 [15]. One of the major functions of cisplatin in cancer therapy is attributed to its ability to suppress the DNA replication of cancer cells [15,16]. However, most questions concerning the treatment of cancer with cisplatin arise from its toxicity, which induces serious side effects, such as gastrotoxicity, myelosuppression, nephrotoxicity, chronic renal failure, etc. [17]. As such, there exists substantial interest in the development of nutraceuticals for combined treatment with chemotherapy, so as to reduce the aforementioned side effects.

Polysaccharides have been widely applied in cancer treatments, for example, polysaccharide-K (Krestin^®^, Kureha Corporation, Tokyo, Japan) from the *Coriolus versicolor* mushroom used in the treatments of colorectal and breast cancers [18,19], and lentinan from the shiitake mushroom used in the treatment of gastric cancer [20]. Antrodan, an immunomodulatory protein-bound polysaccharide prepared from fungal polysaccharides in the mycelia of *Antrodia cinnamomea*, was applied in various biological contexts, including as an anti-inflammatory drug and an antioxidant [21], in the beneficial treatment of benign prostatic hyperplasia [22], and for its protective effects against lipopolysaccharide-induced acute liver damage in rat models [23]. Furthermore, antrodan inhibited the metastasis of Lewis lung carcinomas in vitro, through upregulation of the protein expression of tissue inhibitor of matrix metalloproteinase (TIMP)-1, TIMP-2, and nm23-H1, leading to a decrease in the activities and protein expression of MMP-2 and MMP-9 [24]. We also found that antrodan acted through indirect immunomodulatory effects, by increasing interleukin (IL)-12 and IL-1β levels, and by decreasing tumor necrosis factor alpha (TNF-α), IL-6, and IL-8 levels in experiments using mononuclear cells [24]. However, it was unclear whether antrodan was effective in the inhibition of tumor metastasis in vivo. In this study, Lewis lung carcinoma (LLC) cells, from a highly invasive murine lung cancer cell line, were used to investigate the anti-metastatic activities of antrodan, as well as those in combination with cisplatin, to explore whether antrodan had the potential to act as an adjuvant of cisplatin in anti-cancer therapy.

## 2. Results

### 2.1. Effects of Antrodan Only, Cisplatin Only, and Their Combined Treatment on Body Weight, and Primary Tumor Growth and Metastasis

**Figure 1:**
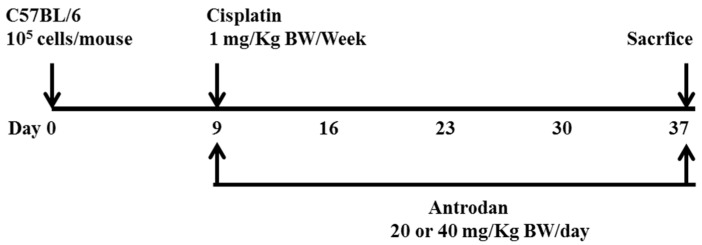
The schedule used for assessing the effects of treatment with antrodan only, and the combined treatment of antrodan with cisplatin in C57BL/6 mice bearing Lewis lung carcinomas (LLCs). LLC cells (10^5^ cells/mouse) were subcutaneously injected into the right flank of each mouse at day 0. Once subcutaneous nodules were visible at the inoculation site (day 9 after inoculation), treatment with antrodan only was orally administered at doses of 20 mg/kg or 40 mg/kg body weight (BW) per day for 28 days. Treatment with cisplatin only was administered at a dose of 1 mg/kg BW, through an intraperitoneal bolus injection, twice per week. Combined treatments of cisplatin (1 mg/kg) with either a low dose (20 mg/kg) or a high dose of antrodan (40 mg/kg) were performed concurrently in the study.

**Figure 2:**
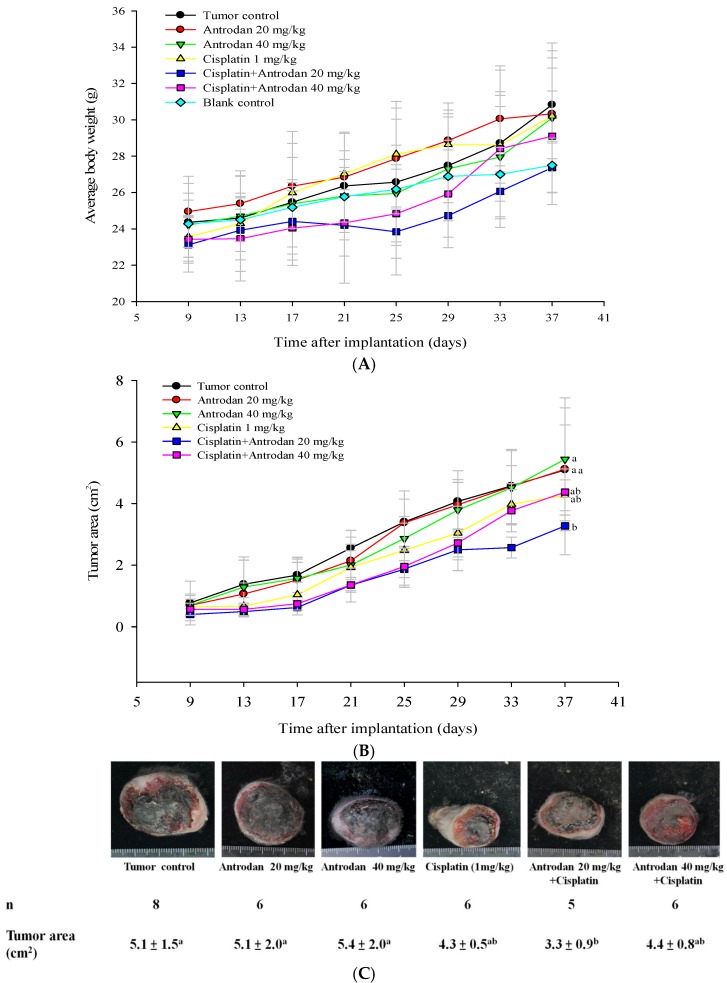
Effects of treatment with antrodan only, cisplatin only, and a combination of both on LLC-bearing C57BL/6 mice. Effect on body weight (**A**), effect on primary tumor area (**B**), and effect on primary tumor morphology (**C**). LLC cells (1 × 10^5^ cells/100 µL) were subcutaneously injected into the right flanks of C57BL/6 mice. Nine days after injection, mice were either administered antrodan (20 mg/40kg and 40 mg/kg; p.o.) daily, cisplatin (1 mg/kg, i.p.) twice per week, or a combination of both for an additional 28 days. Body weight was measured twice per week. The formula for calculating tumor area was: length × width × (π ÷ 4). Means sharing a letter in superscript were not significantly different (*p* > 0.05).

**Figure 3:**
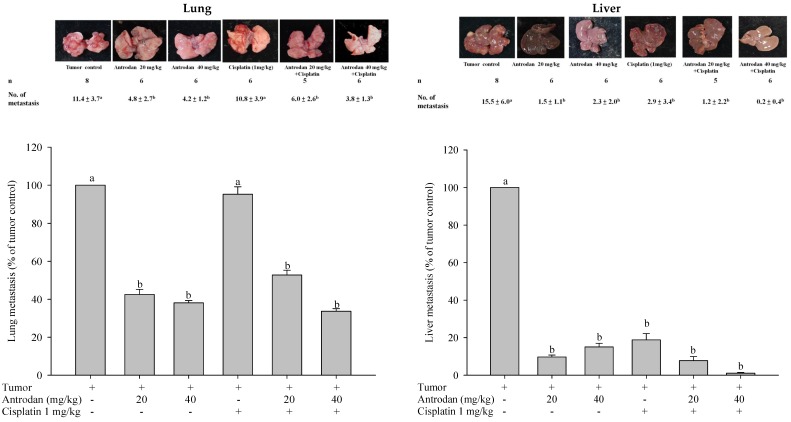
Effects of treatment with antrodan only, cisplatin only, and a combination of both on the metastasis of lung (**left panel**) and liver (**right panel**) foci in LLC-bearing C57BL/6 mice. Percentage metastasis in the tumor control group was 100%. Within each tissue, values not sharing a common letter were significantly different (*p* < 0.05).

To evaluate the effects of antrodan, and its combined treatment with cisplatin on tumor growth and metastasis in LLC-bearing mice, we proposed the experimental schedule shown in Figure 1. When compared with the blank control solution, neither the treatment with antrodan (20 mg/kg and 40 mg/kg), nor the treatment with cisplatin, nor with their combined treatment affected animal growth during the experiment (Figure 2A, Table 1). In addition, there were no significant differences among the group of mice in terms of relative organ weights of livers and kidneys, which were measured after the mice were sacrificed (Table 1). The combined treatment of antrodan (20 mg/kg and 40 mg/kg) with cisplatin significantly reduced the area of the primary tumor, although the reduction in the treatment with 40 mg/kg antrodan was less effective than that in the treatment with 20 mg/kg antrodan, albeit not significantly. Treatment with cisplatin only partially reduced the area of the tumor, but its effect was still lower than that of the combined treatment of antrodan (20 mg/kg) and cisplatin (Figure 2B,C). The results indicated the potential of antrodan as an adjuvant in cancer treatment. Furthermore, no mortality was recorded, and no changes in relative weights of organs were observed in mice treated with antrodan only (Table 1), suggesting a nonadverse impact of antrodan on physiological functions at these doses. In addition, there were no significant differences in the weight ratios of liver to body and of kidney to body among all experimental groups (Table 1). Treatment with antrodan only and its combined treatment with cisplatin resulted in a significant decrease in the number of metastatic foci in lung tissues when compared with tumor control mice, with reductions of 57.9% and 63.2% observed in the treatments with antrodan only (20 mg/kg and 40 mg/kg, respectively), and reductions of 47.4% and 66.7% observed in the combined treatments of antrodan (20 mg/kg and 40 mg/kg, respectively) and cisplatin (Figure 3, left panel). However, treatment with cisplatin only did not affect the metastasis of lung foci. There was significant inhibition of tumor metastasis in liver tissues for all treatment groups. Importantly, the number of metastatic lesions in the liver was significantly reduced (up to 98.7% reduction) when treated with both antrodan (40 mg/kg) and cisplatin (Figure 3, right panel).

### 2.2. Effects of Treatment with Antrodan Only, and Its Combined Treatment with Cisplatin on IL-6 and IFN-γ Levels

Dysregulation of the IL-6 cytokine is one of the biomarkers associated with inflammatory diseases, including cancer [12]. As shown in Table 2, IL-6 levels in the tumor control group were significantly (*p* < 0.05) upregulated to 293 ± 9 pg/mL when compared with levels in the blank control group (135 ± 3 pg/mL). Treatment with antrodan only (20 mg/kg and 40 mg/kg) reduced IL-6 levels to 179 ± 3 pg/mL and 169 ± 38 pg/mL, respectively, which were not significantly different from that of the blank control (Table 2). Treatment with cisplatin only did not reduce IL-6 levels when compared with the tumor control group, whereas IL-6 levels were significantly ameliorated by the treatments with antrodan only. These results suggested that antrodan on its own has a decisive role in regulating the release of the IL-6 cytokine. To further investigate effects of treatment with antrodan on the immune systems of mice inoculated with LLCs, plasma levels of IFN-γ were analyzed through ELISA. The treatment with a high-dose of antrodan only (40 mg/kg) caused a significantly higher increase in plasma IFN-γ levels when compared with that caused by treatments with cisplatin only, and a combination of both (*p* < 0.05) (Table 2). There was no significant difference in plasma IFN-γ levels between both combined treatments of antrodan (20 mg/kg and 40 mg/kg) and cisplatin (1 mg/kg) (Table 2).

### 2.3. Effects of Treatment with Antrodan Only, Cisplatin Only, and a Combination of Both on Protein Activities of MMP-2, MMP-9, and uPA in Plasma of LLC-Bearing Mice

**Figure 4:**
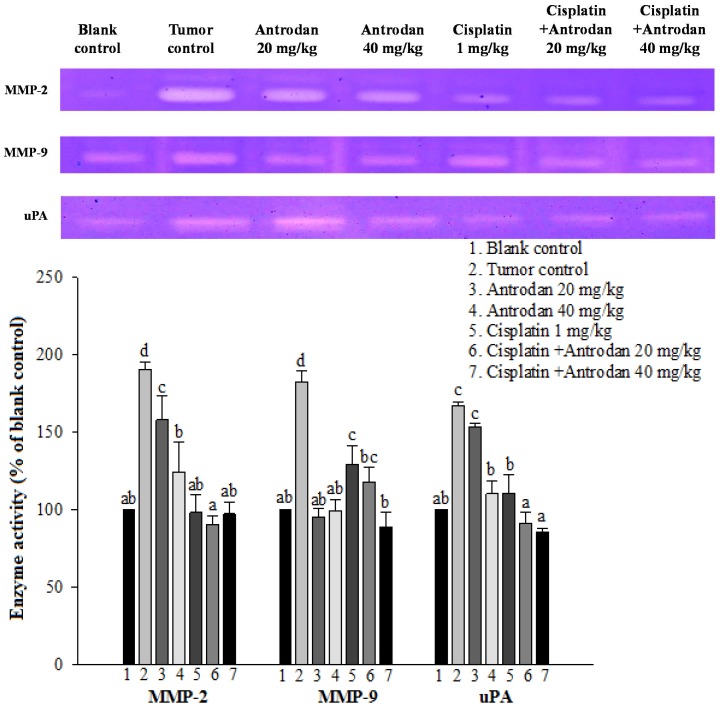
Effects of treatment with antrodan only, cisplatin only, and a combination of both on protein expression of matrix metalloproteinase 2 (MMP-2), MMP-9, and urokinase plasminogen activator (uPA) in plasma of LLC-bearing C57BL/6 mice. Enzyme activity in the blank control group was 100%. Within each enzyme, values not sharing a common letter were significantly different (*p* < 0.05).

The activities of MMP-2, MMP-9, and urokinase plasminogen activator (uPA) in plasma were analyzed through zymography at the end of the experiment. Activities of MMP-2, MMP-9, and uPA in plasma in the tumor control mice were significantly higher than those in the normal control (*p* < 0.05, Figure 4). Treatment with antrodan only at 40 mg/kg significantly reduced the activities of MMP-2 and uPA. Furthermore, treatment with antrodan only at both low and high doses (20 mg/kg and 40 mg/kg) effectively reduced MMP-9 activity to the control level. Treatment with cisplatin only significantly reduced the activities of MMP-9 and uPA, but not that of MMP-2, to normal levels. Interestingly, most of the enzymes’ activities returned to normal levels upon combined treatment with both antrodan and cisplatin (Figure 4).

### 2.4. Effects of Treatment with Antrodan Only, Cisplatin Only, and a Combination of both on Protein Expression of MMP-2/9, STAT3, ERK1/2, JNK1/2, and p38, as well as on Phosphorylation of STAT3, ERK1/2, JNK1/2, and p38 in Lung and Liver Tissues of LLC-Bearing Mice

**Figure 5:**
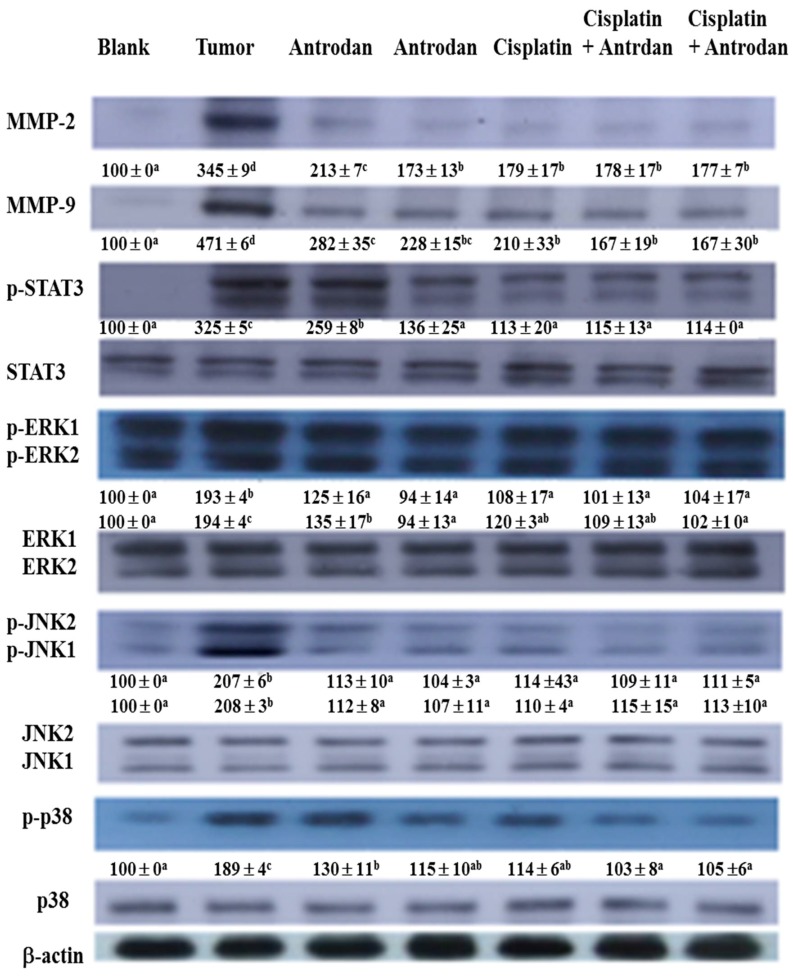
Effects of treatment with antrodan only, cisplatin only, and a combination of both on protein expression of MMP-2 and MMP-9, and on the phosphorylation and protein expression of signal transducer and activator of transcription 3 (STAT3), extracellular signal-regulated kinase 1 (ERK-1), c-Jun N-terminal kinase 1 (JNK1), JNK2, and p38 in lung tissues of C57BL/6 mice. Changes in the levels of each protein were quantified using the AlphaEaseFC software, and relative data were normalized with respect to β-actin levels, and included under each blot, considering the control levels as 100. Within each protein, values with differing letters in superscript were significantly different (*p* < 0.05).

**Figure 6:**
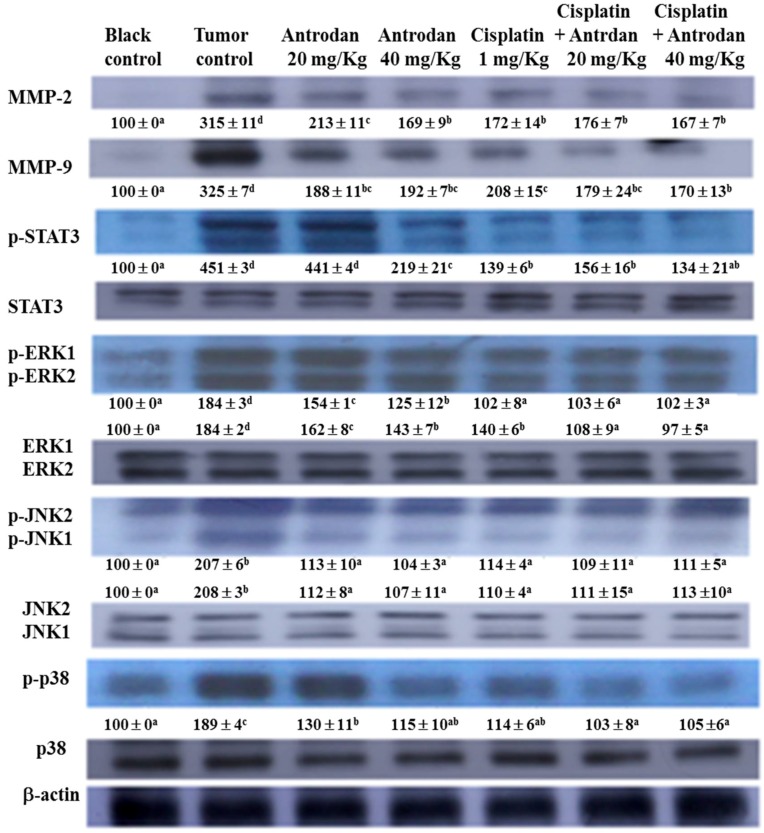
Effects of treatment with antrodan only, cisplatin only, and a combination of both on protein expression of MMP-2 and MMP-9, and on the phosphorylation and protein expression of STAT3, ERK1, JNK1, JNK2, and p38 in liver tissues of C57BL/6 mice. Changes in the levels of each protein were quantified using the AlphaEaseFC software, and relative data were normalized with respect to β-actin levels, and included under each blot, considering the control levels as 100. Within each protein, values with differing letters in superscript were significantly different (*p* < 0.05).

In lung tissues, treatments with antrodan only (20 mg/kg and 40 mg/kg), and cisplatin only (1 mg/kg) significantly reduced protein expression of MMP-2, with reductions of 38.3% (*p* < 0.05), 49.9% (*p* < 0.05), and 48.1% (*p* < 0.05), respectively, when compared with the tumor control group (Figure 4). Similarly, reductions in protein expression of MMP-9 were also seen, with values of 40.1% (*p* < 0.05), 51.6% (*p* < 0.05), and 55.4% (*p* < 0.05), respectively, when compared with the tumor control group (Figure 5). However, the treatments with a combined of both did not further reduce protein expression of MMP-2 and MMP-9, when compared with expression in the treatment with cisplatin only. Interestingly, the treatments with a combination of both significantly (*p* < 0.05) reduced protein expression of MMP-9 in liver tissues (Figure 6).

To explore additional mechanisms of the growth inhibitory effect of treatments with cisplatin and/or antrodan on the metastasis of cancer cells in lung and liver tissues, we analyzed the expression levels of various proteins in both tissues. We determined the ratios of phosphorylated (p) proteins to their unphosphorylated counterparts (pSTAT3 to STAT3, pERK1 to ERK1, pERK2 to ERK2, pJNK1 to JNK1, and pJNK2 to JNK2) so as to evaluate the therapeutic effects on cancer metastasis, using western blotting. We observed a general trend of reduced expression ratios in all of the above signal molecules upon treatments with cisplatin and/or antrodan (Figure 5 and Figure 6). However, a drastic decrease in the ratios of pERK1 to ERK1, and of pERK2 to ERK2 was observed in lung tissues upon treatment with a combination of both. This was also observed in the ratios of pSTAT3 to STAT3, pERK1 to ERK1, and pERK2 to ERK2 in liver tissues. A significant reduction (*p* < 0.05) in the phosphorylation of STAT3 was observed in liver tissues, but not in lung tissues in mice treated with antrodan only, cisplatin only, and a combination of both (Figure 5 and Figure 6), suggesting a specific molecular mechanism involved in liver tissues. Overall, the study indicated that antrodan alone treatment was more efficient in inhibiting metastasis on lung tissues than cisplatin alone treatment (Figure 3, left panel), while the similar inhibition effects of metastasis on liver tissues were found in either antrodan alone or cisplatin alone treatment in LLC-bearing mice (Figure 3, right panel).

### 2.5. Histopathological Findings

**Figure 7:**
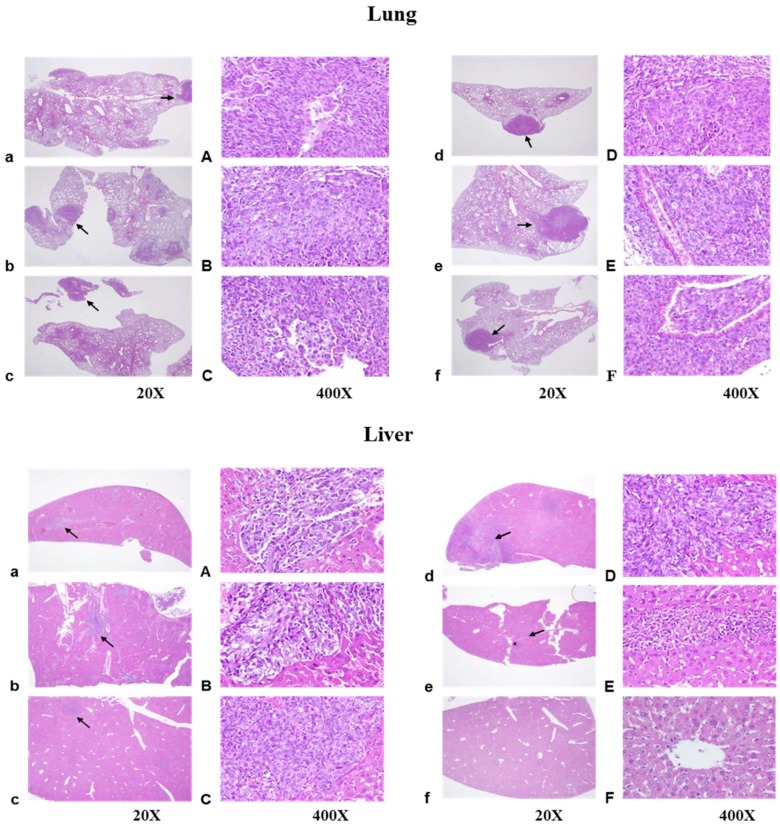
Histological analysis of tumor growth and metastasis of LLC cells in the lung (**upper panel**) and liver (**lower panel**) of C57BL/6 mice after tumor cell implantation and oral administration of antrodan, or i.p. administration of cisplatin. The morphology of multiple, slight to moderate, tumor cell metastases via blood or lymph vessels to the lung and liver in the form of tumor emboli (arrows), which also grew in the parenchyma of the lung, were observed and photographed under the microscope (20× and 400× magnification) in the tumor control group (**a** and **A**), and the groups treated with antrodan only (20 mg/kg, **b** and **B**; 40 mg/kg, **c** and **C**), cisplatin only (1 mg/kg) (**d** and **D**), a combination of antrodan (20 mg/kg) and cisplatin (**e** and **E**), and a combination of antrodan (40 mg/kg) and cisplatin (**f** and **F**). A complete inhibitory effect on liver metastasis was seen upon treatment with a combination of antrodan (40 mg/kg) and cisplatin (**f** and **F**).

To investigate the effects of treatment with antrodan and cisplatin on cancer cell motility and metastasis formation, the lung and liver sections were observed using hematoxylin and eosin (H&E) staining. Figure 7 shows representative images of metastasis in the lung (upper panel) and liver (lower panel). In lung sections, colonization of LLC cells through blood and lymphatic vessels were observed in all groups, including the tumor control group, and the groups treated with antrodan only (20 mg/kg and 40 mg/kg), cisplatin only (1 mg/kg), and combinations of both antrodan (20 mg/kg and 40 mg/kg) and cisplatin (1 mg/kg). Histologic examination of the H&E-stained lung sections showed the formation of tumor emboli (arrows) (Figure 7, upper panel). This was also seen upon examination of H&E-stained liver sections (Figure 7, lower panel), with metastasis of LLC cells observed in most groups. Strikingly, a complete inhibitory effect on the metastasis of LLC cells to liver tissues was achieved through combined treatment with antrodan (40 mg/kg) and cisplatin (Figure 3).

### 2.6. Antrodan Ameliorated Cisplatin-Induced Kidney Dysfunction in LLC-Bearing Mice

**Figure 8:**
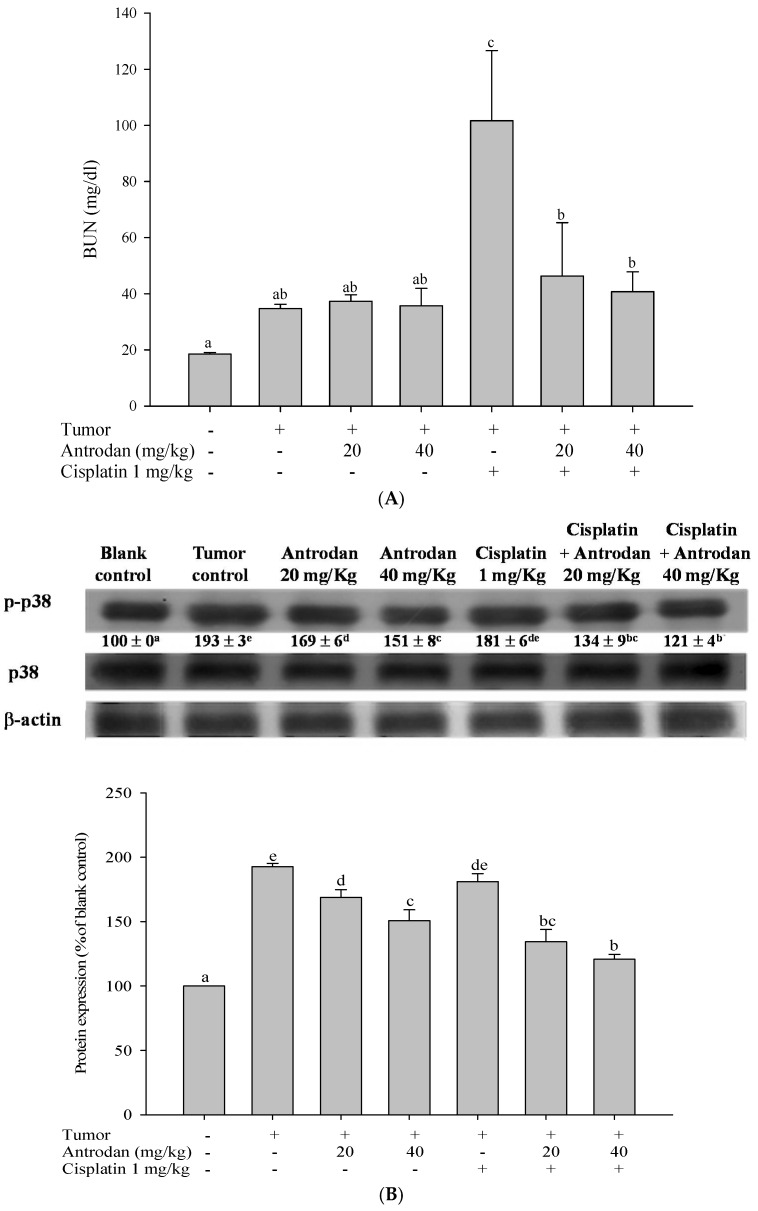
Effects of treatment with antrodan only, cisplatin only, and a combination of both on BUN levels (**A**), and on the phosphorylation and protein expression of p38 in kidney tissues (**B**) in C57BL/6 mice. Western blots of phosphorylated p38 (p-p38), p38, and β-actin (**B-upper**). Densitometric analysis of protein expression (**B-lower**). Changes in the levels of each protein were quantified using the AlphaEaseFC software, and relative data were normalized with respect to β-actin levels, and included under each blot, considering the control levels as 100. In the bar graph, values not sharing a common letter were significantly different (*p* < 0.05). In the western blot, values with differing letters in superscript were significantly different (*p* < 0.05) (**B-upper**).

The presence of cisplatin resulted in significant increases in plasma concentrations of blood urea nitrogen (BUN) (fivefold, *p* < 0.05) (Figure 8A) when compared with normal control groups that received no cisplatin injections. Treatments with a combination of antrodan (20 mg/kg and 40 mg/kg) and cisplatin significantly reduced the levels of cisplatin-induced BUN by ~50% and ~40%, respectively, when compared with treatments with cisplatin only (*p* < 0.05, Figure 8A). We thought that antrodan may have had antioxidant activity, responsible for reducing cisplatin-induced renal oxidative stress in mice [21].

### 2.7. Treatments with Antrodan Only, and Its Combined with Cisplatin Reduced the Phosphorylation of Kidney p38 MAPK

The p38 MAPK is known as a tumor-related signaling molecule, and cisplatin has been proven to induce p38-MAPK activation, resulting in kidney injury [16,17]. In this study, homogenized kidney tissues were used as samples for p38 phosphorylation (p-p38) analysis through western blotting. Results (Figure 8B) indicated that the expression of p-p38 was significantly increased to 193% (*p* < 0.05) in the tumor control group when compared with the normal control group (100%). Treatment with antrodan only (20 mg/kg and 40 mg/kg), but not cisplatin only, significantly (*p* < 0.05) reduced the expression of p-p38 by 13% and 22%, respectively, in comparison with the tumor control group. Furthermore, treatment with a combination of both (antrodan 20 mg/kg, 40 mg/kg + cisplatin 1 mg/kg) dramatically reduced the expression of p-p38 by 30% and 37%, respectively (*p* < 0.05) (Figure 8B).

## 3. Discussion

In this study, we examined the potential of antrodan to both enhance the anti-cancer effects of cisplatin and reduce its side effects in LLC-bearing mice. This study reports, for the first time, that treatment with antrodan only effectively inhibited both lung and liver metastasis, and enhanced the effects of cisplatin on lung metastasis when both agents were combined. Antrodan itself did not inhibit primary tumor growth, nor did it disturb the effect of tumor growth inhibition by cisplatin. Unexpectedly, the effects of treatment with cisplatin only, and the combined treatment of antrodan (40 mg/kg) and cisplatin were less, but not significantly less, than the effect of the combined treatment of antrodan (20 mg/kg) and cisplatin on reduction of the primary tumor area. This non-dose-dependent effect could be attributed to the relatively large variation in tumor area, and the relatively small number (5 to 6) of rats per group. In addition, we found that antrodan effectively prevented cisplatin-induced kidney dysfunction in this animal model.

Possible molecular mechanisms underlying the anti-metastatic effects of antrodan on signal transduction pathways in LLC-bearing mice were elucidated in this study. Firstly, the effects of antrodan were observed on tumor invasion, and metastasis-involved matrix metalloproteinases (MMPs) and serine-proteinases, such as the urokinase plasminogen activator (uPA) [25]. Among the MMPs, MMP-2 and MMP-9 specifically degrade the main structural component of basement membranes [26]. When bound to its receptor, uPA initiates the activation of MMPs. Once activated, MMPs digest the extracellular matrix enzymes (ECMs), therefore facilitating the movement of cancer cells. In addition, overexpression of uPA has been demonstrated to be essential in the maintenance of invasion and metastatic phenotypes [27]. Therefore, inhibition of uPA expression may contribute to a reduction in cancer metastasis. The data presented in Figure 4 appeared to support the findings that the anti-cancer drug—cisplatin—attenuated uPA activity, resulting in a reduction in the expression of MMPs. Unexpectedly, the low-dose treatment with antrodan (20 mg/kg) only induced a higher level of uPA expression, than with the high-dose treatment (40 mg/kg). We supposed that the low-dose treatment with the polysaccharide might have stimulated macrophage activity, and increased the secretion of uPA. Clearly, more work is needed to resolve the molecular requirements and mechanisms for the inhibition of uPA with antrodan. However, the combined treatment of antrodan and cisplatin seemed to better improve the attenuation of uPA activity and the formation of MMPs than treatment with cisplatin only (Figure 4).

IL-6 is an important cytokine that participates in the host’s inflammatory response. IL-6 activates many signaling molecules, including those in the pathways of PI3K, JAK-STAT, and MAPK, which affect the development of cancer. IL-6 promotes cancer cell proliferation and cancer cell metastasis via the PI3K and MAPK pathways. IL-6 also activates STAT-3 to promote VEGF protein expression, angiogenesis, and the growth of cancer cells. In clinical studies, the levels of IL-6 in plasma of cancer patients were positively correlated with the progression of cancer symptoms [28]. Thus, IL-6 is one of the cytokines that has an important role in cancer pathogenesis. In this study, we found that the plasma levels of IL-6 in LLC-bearing mice were significantly reduced by treatment with antrodan only (20 mg/kg and 40 mg/kg), while the levels of IL-6 were not affected by treatment with cisplatin only. These results indicated that antrodan and cisplatin may have different mechanisms of action with respect to inhibiting tumor metastasis. 

IFN-γ inhibits cancer cell growth by promoting innate immune responses, such as the macrophage-mediated killing of cancer cells, antigen presentation, and Th1-type cell activation [29]. In the present study, low doses of antrodan (20 mg/kg) only did not induce a change in IFN-γ concentration in blood plasma of LLC-bearing mice, but higher doses (40 mg/kg) significantly increased plasma concentrations of IFN-γ, superior to those upon treatment with a combination of antrodan and cisplatin (Table 2).

Members of the signal transducer and activator of transcription (STAT) protein family, known as latent cytoplasmic transcription factors, are involved in cell proliferation, differentiation, and survival. STAT3 is activated by many cytokines (e.g., IL-6), growth factors, and the Src and Ras oncoproteins. When STAT-3 is activated, the tumor cell micro-environment is promoted through cell proliferation, angiogenesis, metastasis, and immune evasion [10]. Similarly, STAT-3 overexpression is associated with the metastasis of many types of cancer, including pancreatic cancer, colorectal carcinoma, gastric cancer, and lung cancer [11]. In the present study, we found that treatments with both antrodan only and cisplatin only significantly inhibited STAT-3 phosphorylation in lung and liver tissues. Thus, the evidence suggested that the suppression of tumor metastasis by antrodan was related to the modulation of the IL-6/STAT-3 pathway.

The MAPK family can be divided into three different subclasses, namely extracellular signal-regulated kinases 1 and 2 (ERK-1/2), c-Jun N-terminal kinases (JNKs), and p38 [30]. In cancer cells, ERK/MAPK is activated by the epidermal growth factor receptor, and Ras, leading to the promotion of proliferation, survival, and metastasis of cancer cells [31]. Both JNK/MAPK and p38 MAPK are stress-activated protein kinases that are frequently activated by environmental and genotoxic stress, and inflammatory response factors [30]. JNK/MAPK affects the survival, apoptosis, and metastasis of cancer cells through the regulation of transcription factor AP-1, and the related gene expression of the cell cycle, and through the secretion of MMPs and nuclear hormone receptors (e.g., the retinoic acid receptor). P38 MAPK regulates many physiological functions, including the cell cycle, inflammation, and cell differentiation. Activation of p38 MAPK usually inhibits cell proliferation, but improves proliferation, invasion, inflammation, and angiogenesis in cancer cells. This may be related to the activation of kinases, and the interactions between various pathways [14,32]. Studies have shown that bladder cancer invasion may be promoted by the p38 MAPK pathway through the regulation of the downstream MAPKAPK2 so as to regulate MMP-2 and MMP-9 activity [33]. Herein, we showed that both antrodan and cisplatin significantly inhibited tumor metastasis, and the phosphorylation of ERK1/2, JNK1/2, and p38 in lung and liver tissues. On the other hand, although cisplatin was found to have similar effects, this anti-cancer drug can cause pathological changes, such as renal cell apoptosis and tubular necrosis, leading to an increase in the levels of plasma BUN, and possible acute renal failure [34]. Results of this study showed that the plasma levels of BUN in the tumor control group were significantly higher than those in the blank control group. It was speculated that the rise in levels of BUN and cachexia promotes alienation in tissue proteins [35]. In addition, when compared with the tumor control group, treatment with cisplatin only significantly increased plasma BUN levels; however, this was not observed in treatments with antrodan only or a combination of both. The upregulated levels of BUN through treatment with cisplatin may be ameliorated through treatment with antrodan by reducing or ameliorating the levels of kidney dysfunction and cachexia. In addition, treatment with cisplatin may induce renal cell apoptosis, oxidative stress, and genotoxicity, resulting in kidney damage [16]. The damage mechanism may be related to the promotion of cell death, caused by the activation of ERK, JNK, and p38 MAPK pathways in renal epithelial cells. Among them, p38 MAPK is involved in the production of TNF-α. TNF-α promotes renal oxidative stress, leading to nephrotoxicity. Therefore, inhibition of the phosphorylation of p38 MAPK may represent a potential strategy to alleviate renal dysfunction [36]. When compared with the tumor control group, treatment with antrodan only (20 mg/kg and 40 mg/kg) significantly reduced p38 phosphorylation in the kidney. We hypothesized that the increase of p38 phosphorylation was due to tumor formation in the kidney, and that antrodan can ameliorate kidney damage by reducing the phosphorylation of p38 in the kidney. When compared with the tumor control group, treatment with cisplatin only had no significant effect on the phosphorylation of p38 in the kidney, while the combined treatment of antrodan and cisplatin significantly reduced cisplatin-induced expression of p38, suggesting that the combined treatment could reduce tumor-induced phosphorylation of p38. In this context, our presented data indicated that treatment with antrodan only alleviated cisplatin-induced kidney dysfunction through the p38 M APK pathway.

## 4. Materials and Methods

### 4.1. Chemicals and Antibodies

Dulbecco’s modified eagle medium (DMEM), non-essential amino acids (NEAAs), penicillin, sodium pyruvate, trypsin, and fetal bovine serum (FBS) were obtained from Gibco/BRL (Rockville, MD, USA). Antibodies of mitogen-activated protein kinases (MAPKs), matrix metalloproteinases (MMPs)-2 and -9 were obtained from Cell Signaling (Bevly, MA, USA). Antibodies of signal transducer and activator of transcription 3 (STAT-3), and secondary antibodies of anti-rabbit IgG were obtained from GeneTex (CA, USA). Antibodies of JNK1 (sc-136205), JNK2 (sc-271133), ERK1 (sc-376852), ERK2 (sc-271451), and p38 (sc-398305) were purchased from Santa Cruz (Santa Cruz Biotechnology, Santa Cruz, CA, USA). Cisplatin (50 mg/100 mL) was obtained from Yung Shin (Yung Shin Pharm. Ind. Co., Ltd., Taichung, Taiwan).

### 4.2. Cell Lines

The murine Lewis lung carcinoma (LLC) cells (BCRC NO. 60050) were purchased from the Food Industry Research and Development Institute (FIRDI, Hsinchu, Taiwan), and were cultured in DMEM containing 10% (*v*/*v*) FBS, 0.37% (*w*/*v*) NaHCO_3_, and penicillin (100 unit/mL) in a 37 °C, 5% CO_2_, and 95% air incubator.

### 4.3. Source of Antrodan

Extraction and purification of antrodan from *Antrodia cinnamomea* mycelia was performed as in our previous report [24] with slight modifications. In brief, the freeze-dried and defatted mycelia were dissolved in water (1:10, *w*/*v*), and heated at 80°C for 2 h to remove the water-soluble materials. The residues were then extracted three times with a hot alkaline solution (pH 9.0, 1:10 *w*/*v*) at 80 °C, each time for 2 h. The extracts were combined and filtered. The pH value of the filtrates was adjusted to 4.0 by using 1 M HCl solution, and they were then precipitated overnight at 4 °C. After the precipitates were collected by centrifuging at 3500 *g* for 30 min, they were dialyzed with deionized water (DDW) for three days to remove the free sugars and amino acids (dialysis tube Mw cut-off 12,000–16,000 Da, Wako, Tokyo, Japan), and then freeze-dried to yield base-soluble extracts. The extracts containing antrodan were then loaded onto a Sepharose CL-6B column (3.0 × 82 cm), and eluted with DDW at pH 11.0 (adjusted using 1 M NaOH) to separate polysaccharides at a flow rate of 0.5 mL/min, and to collect the target with a fraction collector. The product was obtained at about 10% yield with an average molecular weight of 442 kD by high-performance size-exclusion chromatography (HPSEC).

### 4.4. Tumor Xenografts and Antrodan/Cisplatin Treatments

Forty-two C57BL/6 male mice (5-weeks-old) were provided by the BioLASCO Taiwan Co., Ltd. (Taipei, Taiwan). All experiments were carried out according to the Animal Research Committee of National Chung Hsing University, and this study was approved by the committee (No. 103-55). The mice were housed in a pathogen-free room at controlled temperature (25 ± 2 °C) and humidity (65 ± 5%), and alternating 12-h-light/-dark cycles. After the mice had been acclimated for one week, 100 µL of saline containing 1 × 10^5^ LLC cells was subcutaneously injected into the right flanks of C57BL/6 mice. Nine days after implantation, the mice with successful tumor formations on the skin were randomly divided into seven groups (*n* = 5–8 for each group) as follows: Group 1, blank control; Group 2, tumor control (implanted LLC cells); Group 3, tumor cell implantation, and oral administration of low-dose antrodan (20 mg/kg); Group 4, tumor cell implantation, and oral administration of high-dose antrodan (40 mg/kg); Group 5, tumor cell implantation, and i.p. administration of cisplatin (1 mg/kg); Group 6, tumor cell implantation, i.p. administration of cisplatin (1 mg/kg), and oral administration of antrodan (20 mg/kg); and Group 7, tumor cell implantation, i.p. administration of cisplatin (1 mg/kg), and oral administration of antrodan (40 mg/kg). The tumor areas were measured once every four days, and calculated as (π ÷ 4) × length (cm) × width (cm) using a vernier caliper [25]. The standard diet (3.3 kcal/g) contained 58.9% carbohydrate, 28.7% protein, and 12.4% crude fat. Antrodan was dissolved in phosphate buffer saline (PBS), and was orally administered once per day, whereas cisplatin (1 mg/kg; i.p., 50 µL) was administered twice per week. For the continuous 28-day treatments, body weight was measured twice per week. The mice were then sacrificed, and the tumors were isolated. Lung, liver, spleen, and kidney samples were harvested for further examination. The number of lung and liver metastases was determined by counting the number of metastatic nodules on the lung and liver surfaces. The lungs and livers were then either stored in the freezer at −80 °C, or fixed with 10% formalin for further studies.

### 4.5. Determination of the Levels of Plasma Cytokines—IL-6 and IFN-γ

Cytokines—interleukin (IL)-6 and IFN-γ—were quantified using commercial kits with solid phase ELISA (R&D systems, Inc., Minneapolis, MN, USA) at a wavelength of 450 nm, performed according to the manufacturer’s protocols.

### 4.6. Determination of MMP-2, MMP-9, and uPA Levels in Plasma through Zymography

Our previous report was followed with respect to the collection and preparation of blood samples [26]. The activities of MMP-2, MMP-9, and uPA in the plasma of mice were measured through gelatin-zymogram protease assays, as previously described [24,37]. In brief, prior to electrophoresis, the prepared plasma was diluted to 1/20 X with PBS and mixed with sodium dodecyl sulfate (SDS) sample loading buffer (5:1, *v*/*v*). Then, the prepared samples were electrophoresed (80 V, 2 h) using 8% sodium dodecyl sulfate (SDS)-polyacrylamide gel electrophoresis (PAGE), containing 0.1% (*w*/*v*) gelatin, and 2% casein-plasminogen. After staining with Coomassie Brilliant Blue R-250, the relative activities of MMP-2, MMP-9, and uPA were quantified through densitometry using the AlphaEaseFC software (Alpha Innotech, Santa Clara, CA, USA).

### 4.7. Western Blotting

Protein expression levels of MMP-2, MMP-9, pSTAT3, STAT3, pERK1, pERK2, ERK1, ERK2, pJNK1, pJNK2, JNK1, JNK2, p-p38, and p38 in lung and liver tissues were measured through western blotting. The lung and liver tissues were homogenized in radio-immunoprecipitation assay (RIPA) buffer and protease inhibitors, and centrifuged (10,000 *g*, 5 min). The supernatants were frozen at −80 °C until ready for use. An amount of protein (40 µg) from the supernatant was mixed with 1/5 X Laemmli sample buffer containing 60 mM Tris-HCl pH 6.8, 25% glycerol, 2% SDS, 14.4 mM β-mercaptoethanol, and 0.1% bromophenol blue, before being denatured by heating to 95 °C for 5 min. Samples were then separated on a 10% SDS-polyacrylamide gel, and electroblotted to nitrocellulose membranes. After blocking with Tris-buffered saline (TBS) buffer (20 mM Tris–HCl, 150 mM NaCl, pH 7.4) containing 5% nonfat milk, the membrane was incubated overnight at 4 °C with the various primary antibodies, followed by horseradish peroxidase-conjugated anti-mouse IgG, and then visualized using an enhanced chemiluminescence ECL detection kit (PerkinElmer, Waltham, MA, USA), and quantified using the AlphaEaseFC software.

### 4.8. Histological Examination of Lung, Liver, and Kidney Tissues

Histological analysis of murine Lewis lung carcinomas in the lung, liver, or kidney of a C57BL/6 mouse was carried out using hematoxylin and eosin (H&E) staining. Tissues were formalin-fixed, embedded in paraffin, 2-μm-sectioned, and then subjected to H&E, and photographed.

### 4.9. Determination of Blood Urea Nitrogen (BUN) Levels

The blood urea nitrogen (BUN) levels were used for analysis of the renal function of mice, and were determined by FUJI DRI-CHEM 3500 (FUJI Technologies, Ebina, Japan). In brief, 20 µL of plasma was prepared as described above, then applied onto the kit, and detected at 625 nm. The BUN concentration was determined by the established calibration curve.

### 4.10. Statistical Analysis

Values were expressed as mean ± standard deviation (SD), and analyzed using one-way ANOVA followed by least significant difference (LSD) for the comparison of group means. All statistical analyses were performed using SPSS for Windows, version 10. Unless specified otherwise, a *p*-value < 0.05 was considered significant.

## 5. Conclusions

**Figure 9:**
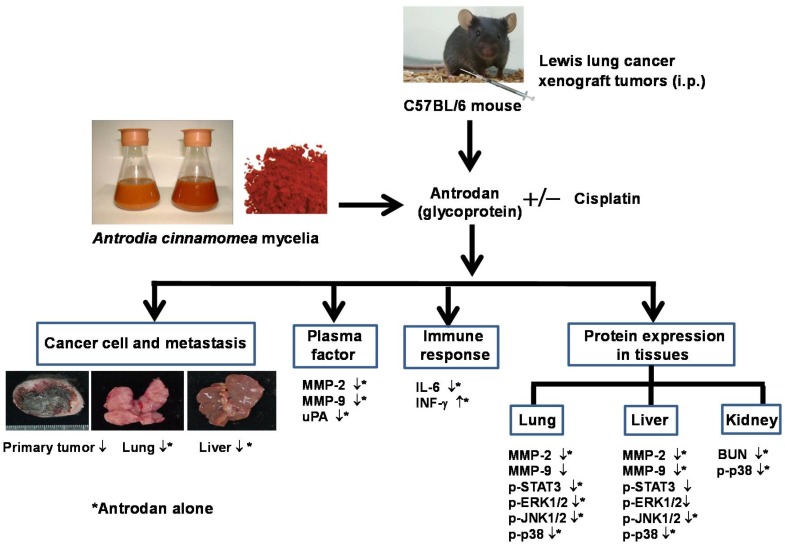
The observed effects of treatment with antrodan only, cisplatin only, and their combination on LLC-bearing C57BL/6 mice in this study. *: Antrodan treatment alone. ↓: Inhibitory effect.

This study showed that antrodan inhibited tumor metastasis rather than the growth of primary tumor cells. Treatment with antrodan only had stronger anti-metastatic potential against tumor metastasis to lung tissues than treatment with cisplatin only in LLC-bearing mice. On the other hand, treatments with antrodan only and cisplatin only were equally effective in inhibiting tumor metastasis to liver tissues. Antrodan apparently inhibited metastasis through several mechanisms, including STAT-3/MAPK/ERK/JNK signaling modulation, immunomodulatory effects, and the amelioration of MMP-2, MMP-9, and uPA factors in plasma, as depicted in Figure 9. These results demonstrated that the therapeutic potential of the novel protein-bound polysaccharide—antrodan—not only enhanced the anti-metastatic effects of cisplatin, but also reduced its side effects on kidney function.

## Figures and Tables

**Table 1:** Effects of treatment with antrodan only, cisplatin only, and a combination of both on C57BL/6 mice bearing Lewis lung carcinomas (LLC)s, in terms of body weight, relative weight, primary tumor weight, and relative organ weight in LLC-bearing C57BL/6 mice ^1^.

Group	n	Body Weight (g)		Relative Body Weight (g) ^2^	Primary Tumor Weight (g)	Relative Weight of Organs ^3^	
		Initial	Final			Liver	Kidney
Blank	6	24.3 ± 1.3 ^a^	27.6 ± 1.5 ^a^	27.6 ± 1.5 ^a^	—	3.7 ± 0.5 ^b,c^	1.2 ± 0.2 ^a^
Tumor control	8	24.4 ± 2.1 ^a^	30.8 ± 3.0 ^a^	20.4 ± 3.6 ^b,c^	10.4 ± 2.5 ^a^	4.5 ± 0.9 ^a,b^	1.0 ± 0.3 ^a^
Antrodan 20 mg/kg	6	24.9 ± 1.9 ^a^	30.3 ± 3.1 ^a^	19.4 ± 2.4 ^c^	10.9 ± 2.4 ^a^	4.0 ± 0.1 ^b^	1.0 ± 0.1 ^a^
Antrodan 40 mg/kg	6	24.2 ± 1.8 ^a^	30.1 ± 4.1 ^a^	19.2 ± 1.0 ^c^	10.1 ± 3.1 ^a,b^	4.3 ± 0.7 ^a,b^	1.1 ± 0.3 ^a^
Cisplatin 1 mg/kg	6	23.6 ± 1.1 ^a^	30.2 ± 1.4 ^a^	23.0 ± 2.0 ^b^	6.8 ± 1.7 ^c^	4.5 ± 0.9 ^a,b^	1.1 ± 0.2 ^a^
Antrodan 20 mg/kg + Cisplatin	5	23.1 ± 1.5 ^a^	27.4 ± 1.4 ^a^	20.4 ± 1.5 ^b,c^	6.9 ± 0.6 ^c^	5.0 ± 0.3 ^a^	1.2 ± 0.1 ^a^
Antrodan 40 mg/kg + Cisplatin	6	23.4 ± 1.3 ^a^	29.1 ± 3.8 ^a^	21.4 ± 3.6 ^b,c^	7.7 ± 0.9 ^b,c^	4.3 ± 0.6 ^a,b^	1.0 ± 0.1 ^a^

^1^ LLC cells (1 × 10^5^ cells/100 µL) were injected (s.c.) once into C57BL/6 mice. After 9 days, mice were treated with either low- or high-dose antrodan (20 mg/kg and 40 mg/kg; p.o.) daily, cisplatin (1 mg/kg, i.p.) twice per week, or a combination of both for an additional 28 days. ^2^ Relative body weights were obtained by subtracting tumor weights from the final body weights (except in the case of those in the blank control group) measured at the end of the experiment. ^3^ Relative weights of organs were calculated as percentages of liver- or kidney-to-body weight ratios. Values are presented as mean ± standard deviation (SD) (*n* = 5–8); means with differing letters in superscript differ significantly (*p* < 0.05).

**Table 2:** Effects of treatment with antrodan only, cisplatin only, and a combination of both on levels of plasma interleukin 6 (IL-6) and IFN-γ ^1^ in LLC-bearing C57BL/6 mice.

Group	*n*	IL-6 (pg/mL)	IFN-γ (pg/mL)
Blank	6	135 ± 30 ^a^	534 ± 221 ^a^
Tumor control	8	293 ± 91 ^b^	2410 ± 450 ^b^
Antrodan 20 mg/kg	6	179 ± 28 ^a^	2562 ± 569 ^b^
Antrodan 40 mg/kg	6	169 ± 38 ^a^	4872 ± 482 ^c^
Cisplatin 1 mg/kg	6	275 ± 84 ^b^	2510 ± 138 ^b^
Antrodan 20 mg/kg + Cisplatin	5	172 ± 18 ^a^	2760 ± 547 ^b^
Antrodan 40 mg/kg + Cisplatin	6	173 ± 36 ^a^	3013 ± 375 ^b^

^1^ LLCs (1 × 10^5^ cells/100 µL) were injected (s.c.) once into C57BL/6 mice. After 9 days, mice were treated with either low- or high-dose antrodan (20 mg/kg and 40 mg/kg; p.o.) daily, cisplatin (1 mg/kg, i.p.) twice per week, or a combination of both for an additional 28 days. Values are presented as mean ± SD (*n* = 5–8); means with differing letters in superscript differ significantly (*p* < 0.05).
